# Correlation between Anterior Cruciate Ligament–Return to Sport after Injury Score at 6 Months after Anterior Cruciate Ligament Reconstruction and Mid-Term Functional Test Results: An Observational Study at 5-Year Follow-Up

**DOI:** 10.3390/jcm13154498

**Published:** 2024-08-01

**Authors:** Alexis Gerfroit, Thibault Marty-Diloy, Pierre Laboudie, Nicolas Graveleau, Nicolas Bouguennec

**Affiliations:** 1 Sports Clinic of Bordeaux-Mérignac, 33700 Mérignac, France; 2 Centre Hospitalo-Universitaire de Poitiers, 86000 Poitiers, France

**Keywords:** anterior cruciate ligament reconstruction, knee rehabilitation, ACL-RSI, return to sport, mid-term results

## Abstract

**Background/Objectives**: Evaluations allowing patients to return to sport (RTS) after anterior cruciate ligament reconstruction (ACLR) should be multimodal, including a psychological evaluation. The goal of this study was to determine if there is a correlation between the ACL–return to sport after injury (ACL-RSI) score at 6 months post-ACLR and mid-term functional results. **Methods**: A total of 498 patients were assessed 6 months after primary ACLR using a composite test including isokinetics, hops, and ACL-RSI. A minimum of 3 years of follow-up was necessary. At the last follow-up, each patient completed clinical and functional evaluations, including the subjective International Knee Documentation Committee (IKDC) score, Tegner Activity Scale, Self Knee Value (SKV), and ACL-RSI score. The results were compared overall and item by item. **Results**: At the last follow-up, the mean SKV, Tegner, IKDC, and ACL-RSI scores were 86.8 ± 14.3%, 6 ± 2.1, 77 ± 11.9%, and 68.8 ± 25.7%, respectively. A significant correlation existed between the 6-month ACL-RSI score and each functional test (respectively, ρ = 0.189 *p <* 0.001; ρ = 0.174 *p <* 0.001; ρ = 0.237 *p <* 0.001). The ACL-RSI score was significantly higher than at 6 months after surgery (*p <* 0.001). Over half (59.2%) of the cohort returned to an equal or greater level of activity, and there was a significant correlation between the 6-month ACL-RSI score and post-surgery level of activity. **Conclusions**: Patients with better ACL-RSI scores at 6 months post-ACLR have better functional results in the medium term and are more likely to RTS. Our results show a correlation between psychological factors at 6 months, measured through the ACL-RSI score, and activity level at mid-term follow-up. This study underlines the relationship between RTS and psychological effects, and the importance of ACLR rehabilitation to focus on decreasing apprehension and fear.

## 1. Introduction

Anterior cruciate ligament reconstruction (ACLR) is one of the most common types of orthopedic surgery [1]. The primary objective of ACLR is to restore the knee’s stability with the goal of a return to sport (RTS). RTS is a crucial point during rehabilitation, even more so with athletes [2]. However, only 56–68% of individuals undergoing ACLR return to a pre-injury level of activity [3,4].

Most studies agree on the variables affecting RTS after ACLR and emphasize the importance of the multimodal aspect of assessment. The evaluation should focus on the function, strength, and power of the quadriceps and hamstrings, hop tests, and any psychological effects [5,6,7,8,9,10].

The psychological aspect is considered more frequently today since an important relationship exists between mental health, physical injury, and subsequent recovery; thus, it is a relevant factor for RTS [9,11,12]. The fear of pain and movement (known as kinesiphobia) after ACLR is one of the main obstacles to RTS [13] and could be associated with the fear of reinjury and/or a lack of confidence [14]. Currently, only a few validated tools allow us to measure this psychological aspect, such as the Tampa Scale [15], specifically designed for kinesiphobia and initially developed for low back pain and fibromyalgia syndrome, or the Injury–Psychological Readiness to Return to Sport (I-PRRS) Scale [16] designed for athletes. The main assessment scale, and the most specific after ACLR, is the Anterior Cruciate Ligament–Return to Sport after Injury (ACL-RSI) scale (Appendix A), described by Webster et al. in 2008 [17]. The ACL-RSI is a global psychological evaluation including emotions (five items), confidence in performance (five items), and risk appraisal (two items), which are associated with the resumption of sport following athletic injury. Recently, Jeon et al. determined a substantial clinical benefit (SCB) value for the ACL-RSI [18] described as a more representative value of the impact of surgery on the daily activities of patients.

There has been some research on the predictivity of functional tests on complication rates and RTS mid-term [19,20]. However, to our knowledge, no studies have investigated the correlation between each test separately, and specifically between the ACL-RSI score at 6 months and functional results at more than 4 years of follow-up.

The aim of this study was to determine whether there is an association between the results of multimodal battery tests at 6 months post-ACLR and functional mid-term performance. Our hypothesis was that a correlation exists between the ACL-RSI score (at 6 months) and mid-term functional results. Our secondary aims were to investigate this association, to search for a correlation between functional results mid-term and each variable at 6 months post-ACLR, and to analyze if patients who reached the SCB value at 6 months were more likely to RTS.

## 2. Materials and Methods

### 2.1. Study Design

This is a retrospective study of data collected prospectively between 2017 and 2020 at the Sports Clinic of Bordeaux–Mérignac (France). It was ethically approved by the appropriate ethics committee and institutional review board of our center (CERC Vivalto Santé; IRB: CERC-VS-01-03-2023). All participants gave their informed consent before inclusion in the study.

### 2.2. Participants

All participants were operated on in the same center by two surgeons who specialized in knee disorders. Patients must have met all of the following inclusion criteria: primary ACLR to avoid confusion in the analysis of outcomes (patients with revision surgery are known to have lower postoperative outcomes [21]); the use of hamstring autografts; and a composite test at 6 months with strength and hop tests plus a psychological evaluation with the ACL-RSI scale. The exclusion criteria were as follows: refusal to participate; secondary ACLR; a graft other than a hamstring autograft; no clinical/functional tests at 6 months post-ACLR or at the last follow-up; less than 3 years of follow-up.

### 2.3. Surgical Technique

The same surgical procedure was used for each patient. ACL reconstruction was performed under arthroscopy using outside-in tibial tunnel drilling, whereas the femoral tunnel was drilled with an inside-out approach. All grafts were 4-strand semitendinosus (4ST) autografts with adjustable cortical endobuttons on the femoral and tibial sides. The menisci were checked and repaired as much as possible (including ramp lesions). Intraoperative findings and treatments were documented in the patient record.

### 2.4. Rehabilitation and Examination

All patients had the same postoperative protocol for physiotherapy (described in Appendix B), with crutches for 1 month, immediate full weight-bearing, and no brace.

The first follow-up with the surgeon at 1 month post-ACLR focused on skin healing, swelling reduction, and recovery of full extension with 90° of flexion.

At 3 months, a second surgical follow-up appointment approved (or not) a progressive return to running and to proprioception work with jump resumption.

During the 6-month appointment, the patient was examined, first by the physiotherapist who conducted the composite tests, including isokinetics, jump tests (Appendix C), and the ACL-RSI scale [6,15], and then by the surgeon with the results of the tests. All assessments were made by three experienced physiotherapists trained in the specific testing protocol used in this study.

Concerning the ACL-RSI scale, a validated French translation was used, with excellent validity and reliability [22]. Each item of the scale was evaluated from 0 to 10 using a mark on a line where the end-points were noted from “extremely” (=0), meaning an extremely negative psychological response, to “not at all” (=10). Then the ACL-RSI score was calculated as follows: ACL-RSI score = (global score × 100)/120 = %. A low final score reflected an important negative psychological impact.

Patients were allowed to RTS by the surgeon between 6 and 12 months post-surgery if there was no pain, no effusion, and a complete range of motion without recurrence of instability, in agreement with the results of the composite evaluation.

At the longest follow-up, patients were examined by a surgeon (not involved in the initial surgery) and asked to fill out Patient-Reported Outcomes (PROs), which comprised the ACL-RSI scale, Self Knee Value (SKV) score, Tegner Activity Scale (TAS), and the International Knee Documentation Committee (IKDC) subjective evaluation form. SKV is a simple and validated functional score that allows an evaluation of the patient with only one question grading the evaluated knee function as a percentage of that of a normal knee (100% meaning normal knee function) [23]. The TAS assesses the knee’s activity from 0 (knee disability) to 10 (national or international athlete) and has already been validated for ACLR [24,25]. Lastly, the subjective IKDC provides a global functional analysis including three categories: symptoms, sports activities, and function [26,27,28]. The results of each item’s score are added and converted to a scale from 0 to 100, where 100 points means a higher level of function or/and a low level of symptoms.

During this last appointment, the patients also answered a questionnaire asking about an RTS and the level of activity. Re-rupture of the graft, contralateral rupture, or new surgery to the knee were also noted.

All data were collected via medical records and stored in our local database.

### 2.5. Outcome Measures

All outcomes of interest were prospectively recorded.

The primary outcome measures were the results at 6 months of the ACL-RSI scale and mid-term functional performance measured with 3 PROMs: the Tegner Activity Scale (TAS), the Self Knee Value (SKV), and IKDC.

The secondary outcomes included the level of activity and RTS at the last appointment. The substantial clinical benefit (SCB) value defined by Jeon et al. [18] was used to search for a correlation between the 6-month ACL-RSI results and RTS/activity level.

### 2.6. Statistical Analyses

The data were summarized using descriptive statistics, including number and percentage for categorical variables, and mean and standard deviation (SD) for continuous variables. The Chi^2^ and Fisher’s exact tests were used to analyze the differences between categorical variables, and Student’s *t* test was used for continuous variables. The data are reported in accordance with the STROBE guidelines [29].

Linear regression was used to determine the significance of correlations between variables. A Shapiro–Wilk test was performed to identify normality and define the adequate statistical procedure. The strength of the correlation was assessed with the Spearman (rho; ρ) correlation coefficient, interpreted as follows: very weak if ρ = 0–0.19, weak if 0.20–0.39, moderate if 0.40–0.59, strong if 0.60–0.79, and very strong if 0.80–1.00. Statistical significance was set at *p <* 0.05. All statistical analyses were performed using IBM SPSS (Statistical Product and Service Solutions) software for Windows (version 27).

## 3. Results

### 3.1. Patient Characteristics

**Figure 1:**
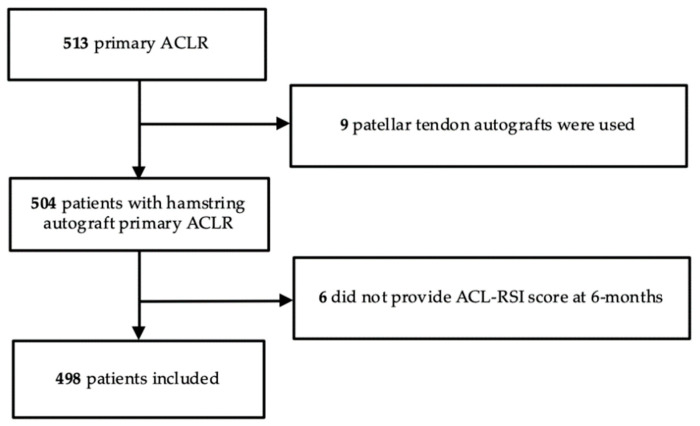
Study flowchart.

During the inclusion period, 504 patients who underwent primary ACLR met the inclusion criteria. Nine patients were excluded because a graft different from a hamstring autograft (patellar tendon) was used for their reconstruction, and six more were excluded because the ACL-RSI score at 6months was missing (Figure 1).

Finally, 498 patients were analyzed: 182 females (36.5%) and 316 males (63.5%), with a mean (SD) follow-up of 4.9 ± 1.23 years. The mean (SD) age at surgery was 27.6 ± 10.9 years (Table 1).

The mean (SD) preoperative Tegner score was 7.1 ± 0.9, corresponding to a competitive sports population for tennis, running, motorcar speedway, and handball, and a recreational sports population for soccer, football, rugby, ice hockey, basketball, squash, and racquetball (Appendix D).

At the last follow-up, 452 patients had been able to RTS (91.1%) and 72.1% (*n* = 359) had resumed the same activity as before the injury: 59.2% returned to the same level of activity or higher (48.8% to the same level, 10.4% to a higher level).

### 3.2. Correlation between 6-Month ACL-RSI Score and Mid-Term Functional Results

The mean (SD) ACL-RSI score was 64.7 ± 38.6% at 6months post-ACLR and 68.8 ± 25.7% at the last appointment, with a significant increase (+4.1%; *p <* 0.001).

There was a significant correlation between the ACL-RSI score at 6months and RTS level (ρ = 0.348; *p <* 0.001) (Table 2). Patients who returned to an equal or higher activity level had a better ACL-RSI score at 6months post-ACLR than those who returned to a lower activity level: the mean (SD) ACL-RSI was 68.7 ± 18.4% vs. 59.2 ± 19.1%, respectively (*p <* 0.001).

Regarding the functional tests at the last follow-up, the mean (SD) results for the SKV, Tegner, and IKDC scores were 86.8 ± 14.3%, 6 ± 2.1, and 77 ± 11.9%, respectively.

There was a significant correlation between the ACL-RSI score at 6 months and each functional test: ρ = 0.189 with SKV score (*p <* 0.001); ρ = 0.174 with Tegner score (*p <* 0.001); and ρ = 0.237 with IKDC score (*p <* 0.001) (Table 2). All functional tests were significantly correlated with each other (Table 2).

Concerning the substantial clinical benefit (SCB), patients who reached an ACL-RSI score of ≥64.2 (SCB value defined by Jeon et al. [18]) at 6 months post-ACLR (*n* = 265) obtained the following averages mid-term: 88.2 for SKV (*p* = 0.024); 6.1 for TAS (*p* = 0.017); 80.6 for IKDC (*p* = 0.0001); and 74.9% for ACL-RSI score (*p* = 0.0001) (Table 3).

Moreover, 92.8% of these patients (*n* = 246) were able to RTS (with 67.5% (*n* = 179) and returned to the same activity level.

## 4. Discussion

The results of this study confirm our hypothesis as there was a correlation between ACL-RSI score at 6 months post-ACLR and mid-term functional results. A higher ACL-RSI score at 6 months was correlated with an equal or higher activity level when patients returned to sport. This difference was a mean of approximately 4 points and was statistically significant in our population of nearly 500 patients at almost 5 years of follow-up.

The majority (91.1%) of the cohort returned to sport, with 72.1% returning to the same sport as before the injury and 59.2% at the same level or higher. The RTS activity level in the literature varies greatly, depending on the pre-injury activity level, age, and the assessment time from surgery. The ACLR surgical technique is seldom taken into account. In a systematic review and meta-analysis from 2014 [5], 55% of the patients returned to competitive sports following ACLR surgery and 65% to the same activity level [5]. Kitaguchi et al. [30] in 2019 reported 81% (101/124 patients) RTS to the same activity level 1 year after ACLR. However, as mentioned by these authors, their better results could be explained by the younger age of the athletes and their very high activity level (mean (SD) age = 17.0 ± 2.7 years; mean pre-injury TAS = 9). Even with a pre-injury TAS of 9, 19% (*n* = 23) of the population did not return to their pre-injury activity level [27]. In 2022, Ueda et al. [31] reported 66% RTS at 1 year, and Cronström et al. [32] achieved 49% RTS to the same activity level at 1 year in 2023.

Our results are similar to those in the literature [33,34,35,36,37] and have the advantage of coming from a homogenous population with well-known characteristics, including only primary ACLR, and where each patient underwent surgery by the same surgical procedure (using hamstring autografts and adjustable fixations).

The composite test was carried out at 6 months post-ACLR, during the re-athletization period. In the literature, several studies, such as those of Blakeney et al., Legnani et al., Müller et al., and Raoul et al., focused on the first 6 months of rehabilitation after ACLR and developed composite tests such as that used by our team [8,38,39,40] with the same purpose: an RTS with optimal performance. The aim of these multimodal battery tests is to identify any deficit to correct it and guide the RTS with objective elements. These tests include hop tests to analyze muscular strength differences and jumping distances between healthy subjects and patients with an ACLR [41,42]. There are also isokinetic tests [43,44] with better reproducibility for concentric exercises and analyses (maximal strength) than eccentric exercises [45,46]. Moreover, Nagai et al. reported a difference in LSI (limb symmetry index) results between hop tests and isokinetic tests for the same patient [47]; these two tests are therefore complementary.

However, in 2020, the Panther Symposium ACL Injury Return to Sport Consensus Group underlined the need for other studies to determine the best way to assess a patient before allowing an RTS [48].

During the 6-month evaluation, the physical examination was completed with a psychological assessment using the ACL-RSI scale [11]. In our study, the mean (SD) ACL-RSI score at 6 months was 64.7 ± 38.6%, which is consistent with the literature [4,49,50]. The ACL-RSI result at 6 months was significantly correlated with the medium-term result for each functional test and with activity level. We also noted that patients who had good ACL-RSI results at 6 months retained better ACL-RSI performance mid-term.

These correlations, in association with the significant increase in ACL-RSI score during follow-up, show that the expected activity level mid-term is set during the first 6 months, which makes this phase of rehabilitation decisive.

Some authors have already come close to these findings. Rosso et al. [51] concluded that a young population (29.5 ± 9.6 years), with an unknown pre-injury activity level, and a low ACL-RSI result (<60) at an average follow-up of 44.1 months, was associated with a lower RTS (OR = 0.04 [95%CI: 0.01–0.21]). Langford et al. [52] observed a correlation between ACL-RSI results at 6 months and RTS rate at 1 year post-ACLR. However, our study is the first to detect this correlation at more than 4 years of follow-up. This result could not be explained by our surgical technique or our rehabilitation protocol because they are not specific to our center and follow the RTS consensus/post-ACLR guidelines. A potential mechanism behind this finding is the length of the rehabilitation period, which was not taken into account in our study, even if we recommend a minimal 6-month rehabilitation period to our patients.

This result should be interpreted with caution, as we already noticed that this is the first study to identify this association at mid-term, and the strength of the correlation is weak (ρ between 0.20–0.39).

Concerning ACL-RSI, several studies have noticed an arbitrary critical value of this score, between 51.3% and 65%, that allows an RTS [8,22,30,52]. For this reason, some authors have tried to determine the most relevant value. In this way, the Minimally Important Change (MIC) was used, measured at 2.6 points (sensitivity: 65%; specificity: 63%) by Slagers et al. [53] on German ACL-RSI. However, this value was only applicable to population averages due to a lack of individual responsiveness, because in their study, only the Smallest Detectable Change (SDC) for groups was smaller than the MIC. According to this study, our significant difference of 4.1 points between 6-month and mid-term ACLR-RSI could be considered as a “real” clinical change. However, the MIC, like the MCID (Minimum Clinically Important Difference), is often considered to be “a floor value” and does not represent clinical success [54]. As such, the Patient-Acceptable Symptom State (PASS), defined by Tubach et al. [55] as the value beyond which patients consider themselves well, could be more relevant, but this value is still missing in the literature for the ACL-RSI scale.

Today, the substantial clinical benefit (SCB) is preferred [8,54] as it is considered to be more representative of the impact on daily activities. The SCB was calculated recently for ACLR by Jeon et al. [18] on a young athletic population (mean (SD) age = 28.6 ± 12.0 years; pre-injury TAS = 8.0 ± 1.5) using two different anchor questions 12 months after ACLR. They determined an ACL-RSI SCB value of 64.2 (anchor question 1, AQ1) with excellent reliability (AUC = 0.92; *p <* 0.001; sensitivity = 81.8%; and specificity = 92.3%) or 57.1 (anchor question 2, AQ2) with poor reliability (AUC = 0.82; *p <* 0.001; sensitivity = 69.6%; and specificity = 84.6%). Using this SCB value of 64.2 points, 53.2% of our population (*n* = 265) reached this threshold at 6 months and 67.5% returned to the same activity level or better in the medium term. Our results support those of Jeon et al., that the ACL-RSI score of patients who attained the SCB at 6 months was significantly improved in the medium term, and was significantly better than the results for patients who did not reach it (Table 3), as with other functional outcomes.

At the last follow-up, three knee function tests were used: SKV, Tegner, and IKDC. Each test was correlated with the activity level recovered, as good function was correlated with better sport resumption. In our results, the pre- and postoperative Tegner scores (7 ± 0.9 and 6 ± 2.1, respectively) were similar to those of previous studies [56], or even higher than in the literature review of Jenny et al. [57]. To the best of our knowledge, SKV has not yet been studied in this population with ACLR, and the mean (SD) result mid-term was 86.8 ± 14%.

The final IKDC score was 77 ± 11.9, corresponding to the results found in the literature mid-term [40,41]. Jeon et al. also calculated the SCB value for this test [18]. They defined the IKDC SCB value as 85.1 (AQ1), or 77.7 (AQ2) with better reliability (AUC = 0.90; *p <* 0.001; sensitivity 95.7%; and specificity 73.1%), and concluded a similarity with the PASS (Patient-Acceptable Symptom State) threshold of 75.9 measured by Muller et al. [8] at a mean follow-up of 3.4 years after ACLR. Both of these are close to our results.

Some of these parameters seem to be more predictive for RTS: the single hop test for distance and the ACL-RSI [8,30,39]. These two elements seem to be protective factors for RTS, and this was confirmed in our study for the ACL-RSI score.

Legnani et al. [39] demonstrated that athletes’ psychological readiness is strictly related to the resumption of jumping and performance. Thus, it is really important for the patient to recommence jumping during the re-athletization period (3–6 months postoperatively).

### Limitations

This study has some limitations and potential biases that need to be acknowledged. First, it is a retrospective study with low- to moderate-quality evidence, like most of the research examining the impact of psychological factors on functional results, as has already been highlighted in the 2016 consensus on RTS [58]. For this reason, future studies should use a prospective design to clarify the impact and develop the temporal relationship between psychological factors and mid-term functional results. Secondly, all the subjects came from the same surgical center, even if the recruitment area was wide (more than 200 km from the clinic), and sample selection bias may have occurred. The use of a questionnaire to investigate the RTS may also have led to detection bias because the results are based on the “good faith” of the patients without ensuring the truthfulness of the answers.

The main strength of this study is the relatively large cohort of patients and the length of follow-up. We also decided to focus on primary ACLR to have a more homogenous population because these athletes are known to be less confident than multi-operated patients [59], and it is not yet known if the responsiveness of the ACL-RSI scale between these two categories is similar (revision vs. first ACLR) [60]. A recent retrospective study found lower psychological readiness results for patients with an ACLR revision [61].

In our study, we only focused on the global ACL-RSI score and did not assess the differences between the different parts of the scale (emotions, confidence in performance, and risk appraisal). Future studies could integrate this distinction, and try to find out which domain is the biggest hurdle for patients.

Each patient followed the same postoperative protocol, but it is not known when they stopped rehabilitation and/or which patients had a psychological intervention during rehabilitation after the first ACL-RSI evaluation. It would be interesting to investigate the impact of this psychological support on RTS and functional results. Moreover, if the mid-term functional results are already defined at 6 months post-ACLR, as the results of our study suggest, this could mean that we should assess the patient’s psychological readiness earlier during the first six months and more frequently because we possibly failed to consider those who obtained poor ACL-RSI results at 6 months. Supervised rehabilitation in a specialized center could be a part of the solution, even if the impact of such rehabilitation on psychological outcomes is still debated compared to home-based rehabilitation.

## 5. Conclusions

Through this study, we compared the results of the composite test carried out 6 months post-ACLR with medium-term activity and function. Our purpose was to highlight any association between the results of these assessments.

We demonstrated that the mid-term functional results and activity levels recovered by these patients are correlated with the ACL-RSI results at 6 months post-ACLR. This is also predictive because the better the ACL-RSI result at 6 months, the better the activity level will be in the medium term. This correlation emphasizes the importance of rehabilitation during the first 6 months post-ACLR and, more specifically, the psychological part. Thus, our primary focus should be psychological rehabilitation and its follow-up to improve the 6-month ACL-RSI results and thereby the mid-term functional results.

## Figures and Tables

**Table 1:** Characteristics of the study population.

Characteristic	Value
Number of patients (*N*)	498
Sex, *n* (%)	
Male	316 (63.5)
Female	182 (36.5)
Age at surgery (years), mean ± SD	27.6 ± 10.9
Follow-up (years), mean ± SD	4.9 ± 1.23
Tegner score (preoperative), mean ± SD	7.1 ± 0.9
ACL-RSI at 6months (%), mean ± SD	64.7 ± 38.6

**Table 2:** Correlation between 6-month ACL-RSI score and functional test results at the last follow-up, and correlation between each functional test.

			ACL-RSI 6M	IKDC	TAS	SKV
Spearman’s Rho	ACL-RSI 6M	Correlation coefficient	1	0.237 **	0.174 **	0.189 **
		Sig (bilateral)		0	0	0
		*N*	498	497	498	498
	IKDC	Correlation coefficient	0.237 **	1	0.345 **	0.661 **
		Sig (bilateral)	0		0	0
		*N*	497	505	505	505
	TAS	Correlation coefficient	0.174 **	0.345 **	1	0.188 **
		Sig (bilateral)	0	0		0
		*N*	498	505	506	506
	SKV	Correlation coefficient	0.189 **	0.661 **	0.188 **	1
		Sig (bilateral)	0	0	0	
		*N*	498	505	506	506

ACL-RSI 6M: Anterior Cruciate Ligament–Return to Sport after Injury score at 6months post-ACLR. IKDC: International Knee Documentation Committee. SKV: Self Knee Value. TAS: Tegner Activity Scale. ** Significant correlation at rank 0.01 (bilaterally).

**Table 3:** Correlation between reaching the SCB at 6 months post-ACLR and medium-term composite test results.

	ACL-RSI ≥ 64.2 (SCB Value) at 6 Months	Patients (*N*=)	Mean	*p* Value
SKV	No	233	85.305	0.024
	Yes	265	88.208	
TAS	No	233	5.732	0.017
	Yes	265	6.174	
IKDC	No	232	76.396	<0.001
	Yes	265	80.602	
Mid-term ACL-RSI	No	233	61.6910	<0.001
	Yes	265	74.9736	

IKDC: International Knee Documentation Committee. SCB: substantial clinical benefit. SKV: Self Knee Value. TAS: Tegner Activity Scale.

## Data Availability

The data that support the findings of this study are available from the corresponding author, Alexis Gerfroit, upon reasonable request.

## Appendix A. Anterior Cruciate Ligament–Return to Sport after Injury (ACL-RSI) Scale

1. Are you confident that you can perform at your previous level of sport participation? 2. Do you think you are likely to re-injure your knee by participating in your sport? 3. Are you nervous about playing your sport? 4. Are you confident that your knee will not give way by playing your sport? 5. Are you confident that you could play your sport without concern for your knee? 6. Do you find it frustrating to have to consider your knee with respect to your sport? 7. Are you fearful of re-injuring your knee by playing your sport? 8. Are you confident about your knee holding up under pressure? 9. Are you afraid of accidentally injuring your knee by playing your sport? 10. Do thoughts of having to go through surgery and rehabilitation prevent you from playing your sport? 11. Are you confident about your ability to perform well at your sport? 12. Do you feel relaxed about playing your sport?
Webster KE, Feller JA, Lambros C. Development and preliminary validation of a scale to measure the psychological impact of returning to sport following anterior cruciate ligament reconstruction surgery. Phys Ther Sport. 2008;9(1):9-15. https://doi.org/10.1016/j.ptsp.2007.09.003 [17] Validated French translation: Bohu, Y., Klouche, S., Lefevre, N. et al. Translation, cross-cultural adaptation and validation of the French version of the Anterior Cruciate Ligament-Return to Sport after Injury (ACL-RSI) scale. Knee Surg Sports Traumatol Arthrosc 23, 1192–1196 (2015). https://doi.org/10.1007/s00167-014-2942-4 [22]

## Appendix B. Steps of the Postoperative Rehabilitation Protocol

**Phase/Stage**	**Description**
First month	Crutches with full weight-bearing, no brace. Rest, icing, compression. Recovering effective quadriceps contractions, full active extension, 90° of flexion
2nd and 3rd months	Recovering flexion to 120° Quadriceps isometric exercises Progressive hamstring work Return to cycling/swimming (no breaststroke) from 5th week
3rd month	Progressive return to running—re-athletization (including jumps)
4th, 5th, and 6th months	Proprioception working (with weight-bearing) Analytic and prevention work to avoid other injury
From 6th months	Progressive return to pivot-contact sports (contact allowed after 8th month) Patient’s sport-specific exercises
Each step has to be completed before moving on to the next one	

## Appendix C. Physiotherapy Protocol 6 Months after Anterior Cruciate Ligament Reconstruction

**Exercises**	**Description**
Warm up	10 min warm up on a cycle ergometer
Isokinetic tests	On isokinetic dynamometer machine (Cybex NORM^®^, Humac, CA, USA): three series of under-maximum effort before each test (familiarization with the exercise, good understanding of the instructions, vocal encouragement): - Three maximum concentric repetitions at 60°/s for strength. Peak torque, peak torque/body weight (PTBW), and flexion-to-extension ratio work were recorded bilaterally and limb symmetry indexes (LSIs) were calculated. - Fifteen maximum concentric repetitions at 240°/s for stamina. Total work, total work/body weight (TWBW), and flexion-to-extension ratio were recorded bilaterally and LSIs were calculated. - Three maximum eccentric repetitions only for hamstring at 30°/s for eccentric strength. Peak torque and maximum peak torque degree were recorded. The LSIs were also calculated. 45 s rest between each exercise.
Hop tests	All hop tests were measured bilaterally. Only successful trials were recorded. The LSI was calculated for all hop tests: - Single-leg squat, movement quality assessment, five repetitions. - Drop jump test, movement quality and maximum distance were measured. Three practice trials were performed. - Single-leg hop test: maximum distance and LSI were measured. Three attempts. - - Triple-leg hop test: maximum distance and LSI were measured. Three practice trials were performed. - - Side-hop test: number of hops performed during 30 s was measured. Five practice trials were performed. 3 to 5 min rest between each jump test and 30 s between each try.
ACLR: anterior cruciate ligament reconstruction; LSI: limb symmetry index; PTBW: peak torque/body weight; SLS: single-leg squat; TWBW: total work/body weight.	

## Appendix D. Tegner Activity Scale

**Level 10**	Competitive sports: soccer, football, rugby (national or international elite)
**Level 9**	Competitive sports: soccer, football, rugby (lower divisions); ice hockey; wrestling; gymnastics; basketball
**Level 8**	Competitive sports: racquetball, squash or badminton, track and field athletics (jumping, etc.), downhill skiing
**Level 7**	Competitive sports: tennis, running, motorcar speedway, handball; recreational sports: soccer, football, rugby, ice hockey, basketball, squash, racquetball, running
**Level 6**	Recreational sports: tennis and badminton, handball, racquetball, downhill skiing, jogging at least five times per week
**Level 5**	Work: heavy labor (construction, etc.); competitive sports: cycling, cross-country skiing; recreational sports: jogging on uneven ground at least twice weekly
**Level 4**	Work: moderately heavy labor (e.g., truck driving, etc.); recreational sports: cycling, cross-country skiing, jogging on even ground at least twice weekly
**Level 3**	Work: light labor (nursing, etc.); competitive and recreational sports: swimming, walking in forest possible
**Level 2**	Walking on uneven ground is possible but impossible to backpack or hike
**Level 1**	Work: sedentary (secretarial, etc.)
**Level 0**	Sick leave or disability pension because of knee problems
Tegner Y, Lysholm J. Rating systems in the evaluation of knee ligament injuries. Clin Orthop Relat Res. 1985;(198):43-49. [62]	
